# *Legionella *species colonization of water distribution systems, pools and air conditioning systems in cruise ships and ferries

**DOI:** 10.1186/1471-2458-8-390

**Published:** 2008-11-24

**Authors:** Georgia Goutziana, Varvara A Mouchtouri, Maria Karanika, Antonios Kavagias, Nikolaos E Stathakis, Kostantinos Gourgoulianis, Jenny Kremastinou, Christos Hadjichristodoulou

**Affiliations:** 1 Department of Hygiene and Epidemiology, Faculty of Medicine, University of Thessaly, Larissa, Greece; 2 Ministry of Mercantile Marine, Piraeus, Greece; 3 Department of Internal Medicine, Faculty of Medicine, University of Thessaly, Larissa, Greece; 4 Department of Respiratory Medicine, Faculty of Medicine, University of Thessaly, University Hospital of Larissa, Larissa, Greece; 5 National School of Public Health, Department of Public and Administrative Health, Athens, Greece

## Abstract

**Background:**

Legionnaires' disease continues to be a public health concern in passenger ships. This study was scheduled in order to investigate *Legionella *spp. colonization of water distribution systems (WDS), recreational pools, and air-conditioning systems on board ferries and cruise ships in an attempt to identify risk factors for *Legionella *spp. colonization associated with ship water systems and water characteristics.

**Methods:**

Water systems of 21 ferries and 10 cruise ships including WDS, air conditioning systems and pools were investigated for the presence of *Legionella *spp.

**Results:**

The 133 samples collected from the 10 cruise ships WDS, air conditioning systems and pools were negative for *Legionella *spp. Of the 21 ferries WDS examined, 14 (66.7%) were legionellae-positive. A total of 276 samples were collected from WDS and air conditioning systems. *Legionella *spp. was isolated from 37.8% of the hot water samples and 17.5% of the cold water samples. Of the total 96 positive isolates, 87 (90.6%) were *L. pneumophila*. *Legionella *spp. colonization was positively associated with ship age. The temperature of the hot water samples was negatively associated with colonization of *L. pneumophila *serogroup (sg) 1 and that of *L. pneumophila *sg 2 to 14. Increases in pH ≥7.8 and total plate count ≥400 CFU/L, correlated positively with the counts of *L. pneumophila *sg 2 to 14 and *Legionella *spp. respectively. Free chlorine of ≥0.2 mg/L inhibited colonization of *Legionella *spp.

**Conclusion:**

WDS of ferries can be heavily colonized by *Legionella *spp. and may present a risk of Legionnaires' disease for passengers and crew members. Guidelines and advising of Legionnaires' disease prevention regarding ferries are needed, in particular for operators and crew members.

## Background

It is well documented that cases of Legionnaires' disease (LD) have occurred among passengers of cruise ships and ferries [1,2]. A World Health Organization compendium published in 2001, showed that from 1970 to 2000, 51 incidents of LD were reported to be associated with ships, involving almost 200 cases, while 10 fatalities were recorded [3]. Since then, repeated reports of outbreaks of LD in passenger ships have been published in the scientific literature [4-6]. In 2006, a total of 11 cases of ship associated LD were reported to the European Working Group for Legionella Infections [7]. From November 2003 through May 2004, eight cases of LD among persons who had recently travelled on cruise ships were reported to the United States Centres for Diseases Control and Prevention [8]. Water distribution systems [9] and whirlpool spas [6,10] of passenger ships have been identified as the source of infection, while a possible link with air-conditioning systems have also been documented [11].

About 30 million passengers are transferred every year by ferries from continental Greece, to the islands. Furthermore, many cruise ships sailing in the Mediterranean Sea include in their itineraries, Greek ports destinations. In 2004, a Belgian passenger travelling by ship from Greece to Italy developed LD [2], while two cases were associated with ferries in 1994 and 1997. However, both patients were exposed to other likely sources of infection (hotels) and no ferries were investigated microbiologically [2]. Few data exists regarding the prevalence of *Legionella *spp. contamination, and the factors that contribute to *Legionella *spp. colonization of water systems in passenger ships, and even fewer data exists for ferries.

The aim of this study was to investigate *Legionella *spp. colonization of water distribution systems (WDS), recreational pools, and air-conditioning systems aboard ferries and cruise ships and to identify risk factors for *Legionella *spp. colonization associated with ship water systems and water characteristics.

## Methods

### Sample collection

From May through July 2004, 269 samples were collected from hot (172 samples) and cold (97 samples) WDS and 7 samples from air conditioning systems of 21 ferries berthed at harbours in Greece. Moreover, 123 samples were collected from hot (103 samples) and cold (20 samples) WDS, 2 samples from air conditioning systems and 8 samples from spa pools of the 10 cruise ships that acted as floating hotels at the Piraeus harbour during the Olympic Games in August 2004. The sample collection and microbiological analysis were part of the Environmental Health Surveillance Program developed by the Olympic Planning Unit and implemented for the Athens 2004 Olympic Games, which is described elsewhere [12]. The sampling was conducted by the Public Health Officers of the Prefecture Public Health Department of Piraeus in cooperation with the National School of Public Health personnel.

In order to obtain a representative sample of each ship WDS, 4 to 20 samples were collected from selected predetermined points, while the total number of samples was based on the total number of cabins and number of decks. The sampling points were chosen according to the EWGLI guidelines [7] a) in the engine room, the hot water leaving the water heater, and when possible the circulating hot water returning to the heater, b) the outlet nearest to the entry of the hot water into the ship WDS, and the most distal site within the WDS, c) showers or taps of crew members or passenger cabins on different decks to be representative of the different loops of the WDS. Four samples were collected from each cabin: One sample was collected from hot water discharging from the shower head immediately after it was turned on (immediate sample) and the second after 60 seconds flow time (post flush sample). Another sample was collected from cold water discharging from the tap immediately after it was turned on and the fourth after two minutes flow time [7]. The microbiological test results of the samples (immediate and "post flush sample") collected from each point (tap or shower head) was considered as a unique result, to characterise the sampling point as contaminated and thereafter in the assessment of the need for remedial actions according to the EWGLI criteria.

Two samples were collected from each swimming or spa pool. In addition, samples collected from the condensate sumps and their drainage arrangements of the ship air conditioning system.

A standardized form was completed during the sampling including details on: sampling point (cabin, fan room, pool), sample temperature, free chlorine, and pH. The temperatures were measured using a calibrated thermometer, placed in the middle of the water stream. Free chlorine and pH were measured using a portable, microprocessor-based meter HANNA HI93710. Samples were collected in 0.5 litre sterile containers containing sufficient sodium thiosulphate to neutralize any chlorine or other oxidizing biocides.

### Microbiological analysis

Water samples were tested for *Legionella *spp. using methods in accordance with ISO 11731 [13], and for aerobic count at 37°C for a minimum of 48 h incubation using the pour-plate method according to ISO 6222 [14]. Microbiologic analysis for *Legionella *spp. was performed by the National Legionella Reference Laboratory of Southern Greece in Athens. The detection limit of *Legionella *was ≥500 CFU/L. Before and during the research period, the reference laboratory participated in an external quality-control scheme for *Legionella *spp. detection in water (Quality Management Ltd, Lancashire, UK), through a periodic distribution of the water samples seeded with unknown *Legionella *spp. and concentration. The total microbial counts at 37°C for 48 h were obtained twice by the pour-plate method on yeast extract agar (LAB M). Methods used for the microbiological analysis have been described elsewhere [15].

### Thermal disinfection

Thermal disinfection was carried out by raising the temperature of the hot water storage tank content to 70–80°C and then circulating this water throughout the system for up to three days. The temperature at the hot water storage tank was raised high enough to ensure that the temperatures at the taps and appliances would not have fallen below 65°C. Each tap and appliance was run sequentially for at least five minutes at the full temperature, and the temperature was measured. The standardized disinfection guidelines were printed and distributed to the persons responsible for the buildings by the Prefecture Departments of Public Health personnel. Hoteliers or the hospital staff applied the disinfection procedure, and when possible supervised by the Prefecture Departments of Public Health personnel [7].

### Risk factors

A detailed standardized questionnaire was developed to record information about the ship water supply systems, which was used to evaluate risk factors, possibly associated with *Legionella *spp. colonization and included information on: 1) the ship characteristics: ship name, unique code, type of ship, owner, itinerary, net tonnage, gross tonnage, ship age, passenger and crew member capacity, 2) the water distribution system components: type of water source (onshore bunkered, evaporator, reverse osmosis), number of water tanks, and presence of water treatment system including its characteristics (disinfection, filtration, softening), 3) the water distribution system characteristics: plumbing material, water tank material, and thermal insulation of the system, and, 4) the heating system, presence of a hot water tank and its volume, and characteristics of water recirculation systems. Water operating temperature and the last date of disinfection was also recorded.

### Statistical analysis

Data were analyzed with Epi-Info 2000 (CDC, Atlanta, GA, USA) and SPSS for Windows Release 11.0.1 software (SPSS Inc, Chicago, IL, USA). Chi square test or Fisher exact test were used for analyzing qualitative data, while t test or Mann-Whitney test were used for quantitative data. Relative risk (RR) and 95% confidence interval (CI) were calculated to assess categorical risk variables associated with legionellae-positive test results. Results were considered statistically significant when the P value was <0.05.

## Results

No sample collected from the cruise ships was positive for *Legionella *spp.

Hot WDS of 14 ferries (66.7%) showed a *Legionella *spp. count ≥500 CFU/L in at least one sample. Hot WDS of 13 ferries (61.9%) fulfilled the criteria of the European Surveillance Scheme for Travel Associated Legionnaires' Disease guidelines (EWGLI) [7] for taking remedial actions: at least one sample with bacteria count ≥10^4 ^CFU/L, or more than two samples with bacteria count >10^3 ^but <10^4 ^CFU/L. These ferries required remedial actions according to the EWGLI. A total of 7 ferries WDS were contaminated with concentrations of ≥10^4 ^CFU/L in at least one sample, whereas 4 of 7 ferries were colonized with ≥10^5 ^CFU/L in at least one sample. Out of the 13 WDS disinfected, 5 (38.5%) needed second disinfection application and two (15.4%) needed third.

A total of 65 hot water samples (37.8%) were colonized by *Legionella *spp. with concentrations of ≥500 CFU/L, while 20 samples had concentrations of *Legionella *spp. ≥10^4 ^CFU/L (Table 1). The greatest colony concentration was 3 × 10^5 ^CFU/L. From the total of 96 positive *Legionella *spp. isolates, 87 (90.6%) were *L. pneumophila*. *L. pneumophila *sg 1 was identified in 44.8% of the total isolates, while *L. pneumophila *sg 2–14 was identified in 45.8% and *Legionella non pneumophila *spp. in 9.4% (Table 1). Samples collected from air conditioning system of ferries were negative for *Legionella *spp. colonization.

**Table 1:** *Legionella *spp. colonization of hot and cold water samples examined from ferries water distribution systems

No. of samples/total (%)	*Legionella *spp.	*L. pneumophila serogroup 1*	*L. pneumophila serogroups 2–14*	*Legionella non pneumophila*
**Hot water samples**				
No. of positive samples/total (%)	65/172 (37.8)	32/172 (18.6)	38/172 (22.1)	7/172 (4.1)
No. of samples with 500–999 CFU/L/total (%)	14/65 (21.5)	9/32 (28.1)	2/38 (5.3)	5/7 (71.4)
No. of samples with 10^3^-9999 CFU/L/total (%)	31/65 (47.7)	19/32 (59.4)	20/38 (52.6)	1/7 (14.3)
No. of samples with ≥ 10^4 ^CFU/L/total (%)	20/65 (30.8)	4/32 (12.5)	16/38 (42.1)	1/7 (14.3)
				
**Cold water samples**				
No. of positive samples/total (%)	17/97 (17.5)	11/97 (11.3)	6/97 (6.2)	2/97 (2.1)
No. of samples with 500–999 CFU/L/total (%)	9/17 (52.9)	7/11 (63.6)	1/6 (16.7)	1/2 (50.0)
No. of samples with 10^3^-9999 CFU/L/total (%)	7/17 (41.2)	4/11 (36.4)	4/6 (66.7)	1/2 (50.0)
No. of samples with ≥ 10^4 ^CFU/L/total (%)	1/17 (5.9)	0/11 (0.0)	1/6 (16.7)	0/2 (0.0)

Out of the 97 cold water samples examined, 17 (17.5%) were positive, 7 samples had concentrations between 10^3^-9999 CFU/L and 1 sample was heavily colonized with colony counts ≥10^4 ^CFU/L (Table 1).

A statistically significant lower water temperature (P = 0.0004) was found in ferries hot water samples [median = 50°C (Interquartile range, IQR = 41.60–55.95)] in comparison to the temperatures found in cruise ships [median = 53.5°C (IQR = 50.00–56.80)]. The median temperature of ferries cold water samples was 23°C (IQR = 20.30–26.00).

*Legionella *colonization was positively associated with ship age (legionellae-negative ferries WDS: median age = 5 years, Interquartile range (IQR) = 3.00–20.00, legionellae-positive ferries WDS: median age = 26 years, IQR = 5.00–31.00, P = 0.02). No association was found with the number of cabins, decks, passenger capacity, itinerary, plumbing material, date of last inspection and last disinfection treatment. The temperature of the hot water samples was negatively associated with colonization of *L. pneumophila serogroup *(sg) 1 (RR = 0.22, P = 0.006) and with *L. pneumophila *sg 2 to 14 (RR = 0.32, P = 0.01). Increases in pH ≥7.8 and total plate count ≥400 CFU/L, correlated positively with the counts of *L. pneumophila *sg 2 to 14 (RR = 2.30, P = 0.005) and *Legionella *spp. (RR = 2.06, P = 0.0002) respectively. No significant association was found between *L. pneumophila *sg 1 concentrations and free chlorine concentration of ≥0.2 mg/L. Contrary, negative significant association was found between free chlorine concentration of ≥0.2 mg/L and samples which were contaminated with *L. pneumophila *sg 2–14, *Legionella *non *pneumophila *and *Legionella *spp. (Table 2).

**Table 2:** Association of physical, chemical, and microbiological water characteristics with *Legionella *spp. concentration of hot and cold water samples on ferries water distribution systems

	**No of positive samples for *Legionella *spp.**							
	**Water characteristics**							
***Legionella *spp.**	**pH**				**Sample Temp**			
	**pH ≥7.8 (%)**	**pH <7.8 (%)**	**RR**	**P**	**≥ 55°C (%)**	**< 55°C (%)**	**RR**	**P**
*Legionella *spp.	32 (32.0)	40 (27.2)	1.17	0.25	7 (14.9)	74 (35.7)	0.41	0. 003
*L. pneumophila *sg 1	17 (17.0)	25 (17.0)	0.99	0.5	2 (4.3)	40 (19.3)	0.22	0.006
*L. pneumophila *sg 2–14	22 (22.0)	14 (9.5)	2.30	0.005	3 (6.4)	41 (19.8)	0.32	0.01
*Legionella *non *pneumophila*	1 (1.0)	7 (4.8)	0.21	0.09	2 (4.3)	7 (3.4)	1.25	0.5
								
	**Free chlorine**				**Total plate count**			
	**≥ 0.2 mg/L (%)**	**< 0.2 mg/L (%)**	**RR**	**P**	**≥400 CFU/L (%)**	**<400 CFU/L (%)**	**RR**	**P**
*Legionella *spp.	8 (12.5)	10 (34.5)	0.36	0.01	26 (53.1)	56 (25.7)	2.09	0.0002
*L. pneumophila *sg 1	6 (9.4)	6 (20.7)	0.45	0.1	13 (26.5)	30 (13.8)	1.92	0.02
*L. pneumophila *sg 2–14	2 (3.1)	4 (13.8)	0.22	0.07	13 (26.9)	31 (14.2)	1.86	0.03
*Legionella *non *pneumophila*	0 (0.0)	2 (6.9)	0.00	0.09	3 (6.1)	6 (2.8)	2.22	0.2

Out of the 101 immediate samples examined, 41 (40.6%) were positive for *Legionella *spp. while, out of the 168 post flush samples examined, 41 (24.4%) were positive (RR = 1.66, CI = 1.17–2.37, P = 0.007). In addition, out of the 139 samples collected from the most distal sites within the WDS, 48 (34.5%) were positive for *Legionella *spp. and out of the 30 samples collected from the outlet nearest to the entry of the hot water into the ship WDS, 15 (50%) were positive for *Legionella *spp. (RR = 1.44, CI = 0.94–2.21, P = 0.08).

## Discussion

Our data indicate that at the time of the study, 66.7% of the water distribution systems of ferries were positive for *Legionella *spp. with concentrations of ≥500 CFU/L in at least one sample and 33.3% were heavily colonized. Extremely high concentrations (>3 × 10^5 ^CFU/L) were found in one ferry and specifically in samples collected from the third class cabins with common showers that were rarely used by passengers. According to the European Surveillance Scheme for Travel Associated Legionnaires' Disease guidelines [7] concerning action levels following *Legionella *sampling in hot and cold water systems, 13 ferries hot water distribution systems (61.9%) needed remedial actions and thermal disinfection was applied. About 38% of the WDS disinfected needed second disinfection application and 15% needed third. This shows that thermal disinfection is not totally effective in eradicating *Legionella *spp. Similar were the findings of a previous study conducted in WDS of buildings (hotels, hospitals etc.) [16].

A similar prevalence study was carried out on 2 cruise ships and 7 ferries docked at the seaports of northern Sardinia in 2004, indicated that 6/7 ferries were positive, 42% (38/90) of the water samples examined were contaminated by *Legionella *spp. and 77.8% of the water samples contained ≥10^4 ^CFU/L [17]. In 1993, a survey carried out in 33 water tanks of yachts in Athens showed that approximately 40% of them were positive for *Legionella *spp. [18]. In our study, we observed a lower frequency of *L. pneumophila *sg 1 (in 43 samples), while 87 of the total 96 isolates from the cold and hot water samples were *L. pneumophila*. In the study carried out in the northern Sardinia, no sample contained *L. pneumophila *sg 1, however *L. pneumophila *was isolated in 42/44 samples (95.5%), followed by *L. micdadei *(4.5%). The strains that were identified in that particular study were *L. pneumophila *sg 6 (45.2%; 19 samples), sg 2 to 14 (42.9%), sg 5 (7.1%) and sg 3 (4.8%) [18].

We observed high colonization (17.5%) of *Legionella *spp. in the cold WDS, compared to the contamination of other buildings such as hotels, where only 6.4% of the cold samples were positive [19]. This may be attributed to the relatively high temperature (median = 23°C, IQR = 20.30–26.00) of cold water aboard ferries. Pipes of cold water pass through the high temperature engine rooms of the ships. Controlling the water temperature in order to prevent *Legionella *spp. colonization of WDS will be effective only if pipes are insulated. Cold water should be maintained at temperatures below [7]. Where this temperature cannot be achieved due to local conditions, suitable alternative residual disinfection procedures must be used and supported by regular (at least quarterly) testing for legionellae [7].

In a previous study aboard 2 cruise ships no water system including potable water distribution system, spa or swimming pool, was positive for *Legionella *spp. [17]. In our study, the relatively high temperature of hot water aboard cruise ships in comparison to the temperatures found in ferries, may partially explain the great difference in *Legionella *spp. colonization between cruise ships and ferries. In addition, the ten cruise ships that were investigated applied the requirements of the United States Vessel Sanitation Program Manual (VSP) regarding potable water safety and swimming and spa pool management. The legionellae-negative microbiological results of the samples collected from the cruise ships indicate that correct compliance with the requirements that were included in the VSP manual were effective to maintain legionellae-free water systems. These guidelines involve continuous halogenation to at least 2.0 mg/L free residual halogen at the time of bunkering or production of potable water, maintenance of a free residual halogen concentration of ≥0.2 mg/L and ≤5.0 mg/L throughout the distribution system and periodical disinfection of the distribution system and water tanks, and other control measures regarding spa pools [20]. Other additional measures may have been applied aboard the cruise ships.

No significant association was found between *L. pneumophila *sg 1 concentrations and free chlorine concentration of ≥0.2 mg/L. Contrary, free chlorine of ≥0.2 mg/L inhibited colonization of *L. pneumophila *sg 2–14, *Legionella *non *pneumophila *and *Legionella *spp. These findings may imply that *L. pneumophila *sg 1 is more resistant in higher chlorine concentrations. This was also observed in a another study in hotels [21].

The high levels of *Legionella *spp. and the high number of positive samples for *L. pneumophila *found aboard ferries, present a risk for infection, since most ship associated cases have been ascribed to infection by *L. pneumophila *sg 1 [2]. However, *L. pneumophila *sg 5 [5,6], 3, 4, and *L. longbeachae *sg 1 [7] have been isolated from clinical specimens of passengers with LD. Unfortunately, we did not monitor the development of pneumonia symptoms among passengers and crew members during the study period. Practical difficulties such as the short stay period aboard ferries ranging from a few hours to one day maximum, limits the evidence for linking the source of infection. In addition, many of the passengers are travelling and may be exposed to legionellae in other locales as they travel (e.g., hotels, restaurants, etc.). However, the limited stay period aboard ferries limits the risk of infection.

Since water systems of ferries could be a possible source for infection, during LD case investigation, exposure history should include information on travelling by ships. This will help to identify passenger cases that usually go undetected, to timely implement environmental control measures and to prevent possible future outbreaks in subsequent voyages.

The temperatures of the hot water samples were negatively associated with contamination with *L. pneumophila *sg 1 and with sg 2 to 14. Similar were the findings of a previous study in hotels WDS [19]. In our study, increases in pH correlated positively with the counts of *L. pneumophila *serogroups 2 to 14. These findings confirm other reports, which indicated a positive association of *L. pneumophila *with pH [19].

The limited number of legionellae-positive WDS of ferries (only 14 positive of the total 21 examined), influence the results of association between colonization and ship factors such as capacity and number of passengers, and therefore we could not draw any conclusions.

LD outbreaks have occurred aboard passenger ships in recent years [8,22], and even fatal cases have been reported [8]. In many cases the source of the organism was clearly demonstrated [9,10]to be the ship's water system. LD outbreaks cause negative economic impact on the cruise ship industry and may result in removing a vessel from service, or delay the sailing from a port to allow control measures to be carried out [2]. Three ships are known to have changed owners and names after being linked with cases of legionellosis [2]. During 2004, there were 205 cruise ships with itineraries in Europe, which represents 87% of all cruise ships in operation . Measures for the prevention and control of the disease should be further taken to a European or even to an International level. In 2005, the European Commission funded the SHIPSAN project which aims at assessing the usefulness of an EU ship sanitation program and coordinated action for the control of communicable diseases in cruise ships and ferries . We believe that a future European ship sanitation program should include LD prevention and control guidelines.

Apart from the EWGLI guidelines which apply in accommodation sites in general, specific measures for passenger ships have been published by the WHO in the revised draft for review of the Guide to Ship Sanitation and in the book entitled "Legionella and the prevention of Legionellosis" [23]. CDC published recommendations to minimize transmission of Legionnaires' disease from whirlpool spas on cruise ship in 1997 [24].

## Conclusion

This study demonstrates that water distribution systems of ferries can be colonized by *Legionella *spp. and present a possible risk for Legionnaires' disease. Both hot and cold water distribution systems can be colonized. Chemical treatment and monitoring of drinking water quality including halogen disinfection, pH adjustment, and water temperature control in hot water systems are recommended as control measures aboard ferries. Older ferries are of higher risk. During LD case investigation, patient history should record any exposure to recent travel by ships. Guidelines and advising of Legionnaires' disease prevention to ferries operators and crew members are needed.

## Competing interests

The authors declare that they have no competing interests.

## Authors' contributions

GG participated in the design of the study, carried out the data collection and sampling. VM participated in the design of the study, carried out the data collection and sampling and drafted the manuscript. MK contributed to the data collection and sampling and helped to draft the manuscript. AK participated in the design of the study. NS, KG and JK participated in the design and coordination of the study. CH conceived of the study, and participated in its design and coordination.

## Pre-publication history

The pre-publication history for this paper can be accessed here:


